# Vision recovery after ocular massage for cosmetic filler-induced ophthalmic artery occlusion

**DOI:** 10.1016/j.ajoc.2024.102229

**Published:** 2024-12-03

**Authors:** Alexander A. Svoronos, Nathan L. Scott

**Affiliations:** Shiley Eye Institute, University of California San Diego, San Diego, CA, USA

## Abstract

**Purpose:**

To report a case of vision recovery after ocular massage for cosmetic filler-induced ophthalmic artery occlusion.

**Observations:**

A 58-year-old female experienced acute loss of vision of the left eye, left ptosis, and left glabellar skin discoloration immediately after cosmetic filler injection, suggestive of occlusion of the branches of the ophthalmic artery. Highly aggressive, prolonged ocular massage was initiated soon after and followed by a substantial recovery of vision. In the following days, signs of anterior segment ischemia and choroidal infarcts developed. Additionally, extramacular preretinal hemorrhages emerged, presumably from shearing of retinal capillaries during the ocular massage. These findings resolved without significant permanent visual deficits.

**Conclusions and Importance:**

We report a unique case of vision recovery after ocular massage in the setting of arterial occlusion due to cosmetic filler injection. It is also a rare case of preretinal hemorrhages associated with ocular massage. The case suggests that ocular massage might, at least in rare circumstances, be effective for treating cosmetic filler-induced retinal artery occlusion.

## Claim of priority

After conducting a literature review on 1/18/24 utilizing PubMed and Google Scholar using the key words (“filler injection”, “artery occlusion”, and “massage” together), we did not find any prior reports of vision recovery due to ocular massage after cosmetic filler-induced ophthalmic or retinal artery occlusion.

In addition, after conducting a literature review on 1/18/24 utilizing PubMed and Google Scholar using the key words (“preretinal hemorrhage ocular massage”) and reviewing all articles that cited the article Uchida et al., Case Rep Ophthalmology, 2015, we did not find any other reports besides Uchida et al., 2015 which described a “valsava-like retinopathy” due to ocular massage.

## 1 Introduction

A rare but potentially devastating complication of cosmetic filler injection is retinal artery occlusion. This can occur if filler enters the arterial circulation and refluxes up to the ophthalmic artery, where it forms an occlusion either at the ophthalmic artery or its more distal branches, including the central retinal artery, resulting in ischemia and vision loss. While no treatments have proven effective in restoring vision after ophthalmic or central retinal artery occlusion, there are anecdotal case reports of improvement of vision after ocular massage. The pressure fluctuations from ocular massage can presumably result in mechanical breakup and/or displacement of the embolus to a more distal vessel, thereby allowing reperfusion of the central retina. In addition, aqueous outflow may be increased during ocular massage, resulting in acutely reduced intraocular pressure and improved ocular perfusion pressure after the massage is complete. Nevertheless, the efficacy of this technique is highly questionable, and, to our knowledge, it has not previously been reported successful for arterial occlusion resulting from cosmetic filler injection. Here, we describe a unique case of vision recovery after ocular massage in a patient with cosmetic filler-induced ophthalmic artery occlusion.

## 2 Case report

**Fig. 1:**
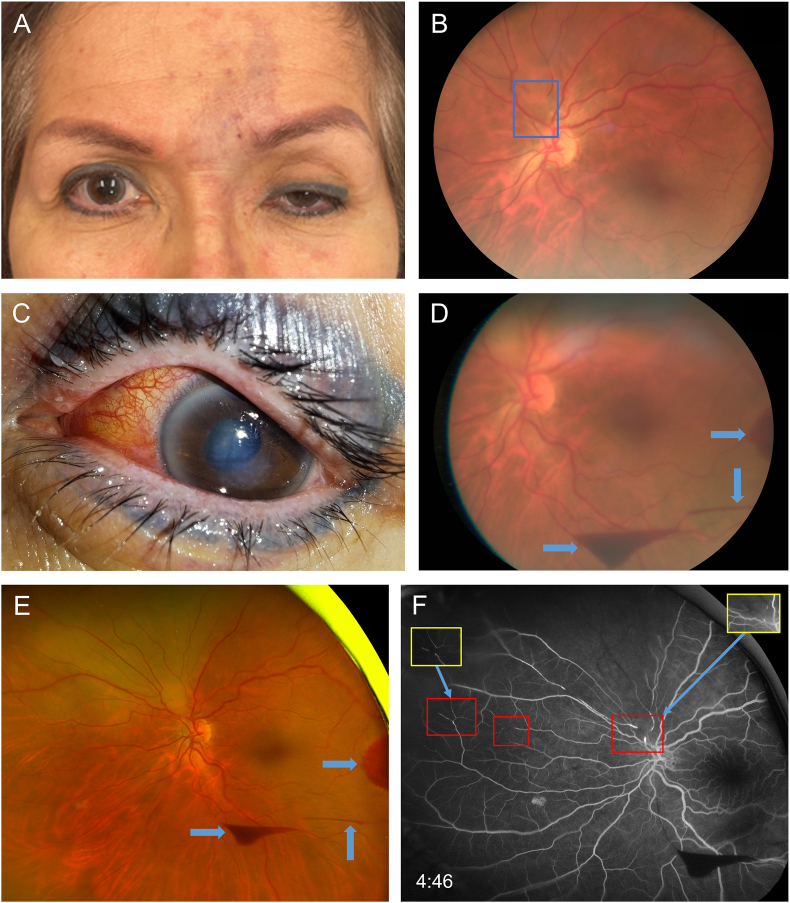
**Exam and imaging findings.** (A) External photo showing purple discoloration of the forehead in the left glabellar region and left-sided ptosis. (B) Fundus photo after ocular massage. A large plaque superonasal to the disc is highlighted by the blue box. (C) External photo of the left eye 3 days after presentation demonstrating conjunctival injection, corneal edema/Descemet's folds, and an irregular pupil, altogether suggestive of anterior segment ischemia. (D) Fundus photo 3 days after presentation and (E) Optos widefield photo 4 days after presentation showing the development of superonasal retinal whitening and preretinal hemorrhages (blue arrows). (F) Fluorescein angiogram demonstrating multiple occlusions, highlighted by the red boxes. The insets bordered in yellow show the corresponding areas 1 minute, 41 seconds earlier, when additional occlusions were apparent. (For interpretation of the references to color in this figure legend, the reader is referred to the Web version of this article.)

A 58-year-old female with no significant past medical history presented to the emergency department with sudden loss of vision in her left eye immediately following a cosmetic filler injection of hyaluronic acid in the left glabellar region approximately 1.5 hours prior to ophthalmic evaluation. The aesthetic physician who injected the filler (RHA® 2 filler, 0.4 mL) additionally observed the patient to have left eye ptosis and purple discoloration of the forehead in the left glabellar region immediately after the injection. Concerned about the possibility of ischemia, the practitioner promptly, within minutes, administered 4 mL of hyaluronidase to the left glabellum and contacted emergency services, who subsequently brought the patient to the emergency department. Upon examination, the patient initially stated that her left eye's vision was significantly blurry with a large dark/black spot in her central visual field. Visual acuity was found to be hand-motion, and she had a mid-dilated and minimally reactive left pupil. Reverse testing revealed a left relative afferent pupillary defect. Intraocular pressure was normal at 16 mmHg. Together with the patient's left ptosis and glabellar skin discoloration (Fig. 1a), a diagnosis of cosmetic filler-induced ophthalmic artery occlusion was made.

Aggressive, continuous ocular massage was immediately initiated after mydriatic eye drops were administered. This involved using two fingers to compress the eye with enough pressure such that the globe was indented/pushed back ∼5 mm within its socket while avoiding pain, and simultaneously employing rapid circular movements to massage the eye with a frequency of roughly two complete circular movements per second. This massage technique was initially performed by the examiner and later by the patient herself, with minimal interruption and no breaks lasting longer than a few seconds. Approximately 15 minutes later, the patient reported a dramatic improvement in her visual acuity and that the central scotoma in her vision had resolved. Upon performing a dilated fundus exam, the presence of a large translucent white plaque in an artery leaving the disc superonasally was identified (Fig. 1b). There was no cherry-red spot or macular pallor, suggesting that her central retinal artery occlusion (CRAO) had possibly moved distally to become a branch retinal artery occlusion (BRAO). Visual acuity was assessed to be at least count-fingers, but more precise testing with a Rosenblaum near card or Snellen chart was not completed, as transportation had arrived to rush the patient for emergent hyperbaric oxygen therapy (of note, the patient did express greatly improved vision prior to transportation, and her vision is believed to have been significantly better than count-fingers at this time). The patient was reassessed a few hours later, after her hyperbaric oxygen therapy (100 % oxygen at 2.8 ATA for 80 minutes) was completed. However, she reported no noticeable improvement in visual acuity compared to immediately after the ocular massage, prior to the hyperbaric treatment. At this point, formalized visual acuity testing with a Rosenblaum near card was performed, which demonstrated her left eye visual acuity to be 20/100, pinhole 20/40. Extraocular movements were full, Ishihara color plates were 8/8, and confrontational visual fields were full to finger-count. Right eye vision was unaffected by the above events and remained at its baseline of 20/40, pinhole 20/25. Blood pressure measurements before and after massage remained approximately constant at 130–140s/60-70s. The patient was subsequently admitted, and hyperbaric oxygen treatments (100 % oxygen at 2.4 ATA for 90 minutes) were planned for twice daily for 3–4 more days. Notably, these treatment parameters were consistent with those used in case reports on hyperbaric oxygen therapy for CRAO found in the literature.^1^, ^2^, ^3^

The following day, the vision was stable, but the patient's left ptosis had worsened, and she developed conjunctival injection of the left eye. Over the following two days, her conjunctival injection increased, she developed corneal edema and Descemet's folds, and her left pupil was noted to be irregular and minimally reactive (Fig. 1c). Altogether, these findings suggested that she had developed anterior segment ischemia, as has previously been reported in other cases of ophthalmic artery occlusion from filler injection.^4^^,^^5^ Visual acuity was correspondingly reduced to 20/200, pinhole 20/100 (likely a result of the aforementioned anterior segment pathology). In addition, her ptosis had further progressed to complete ptosis. Dilated fundus exam (Fig. 1d) now exhibited superonasal retinal whitening distal to the previously noted occluded vessel, confirming the diagnosis of BRAO. In addition, multiple extramacular preretinal hemorrhages were observed. These are believed to have been secondary to a “valsava-like retinopathy” induced by ocular massage, as had once been reported previously.^6^ The patient was discharged the following day for further management as an outpatient, including ∼1 month of daily (excluding weekends) hyperbaric oxygen treatments.

Fluorescein angiography was conducted on the day of discharge and demonstrated superonasal nonperfusion distal to the previously noted occlusion (Fig. 1f). In addition, multiple other, smaller and more distal occlusions were noted in separate arteries, suggesting that the original hyaluronic acid plaque had broken up into several smaller pieces, which had traveled down separate arteries to cause multiple small BRAOs in addition to the large superonasal BRAO. A macular OCT was normal, and Optos ultra-widefield images redemonstrated the superonasal retinal whitening and extramacular preretinal hemorrhages (Fig. 1e).

**Fig. 2:**
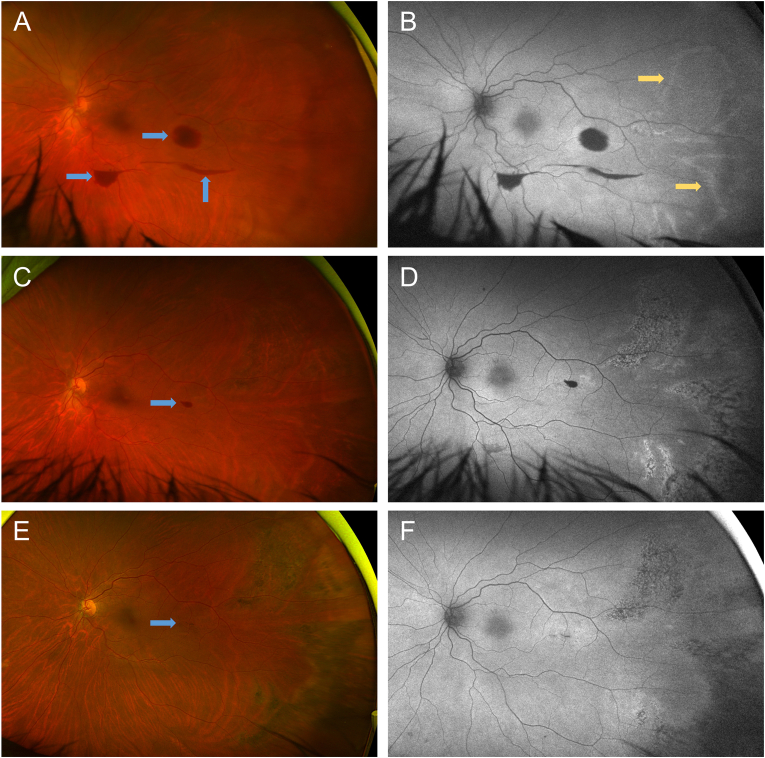
**Outpatient Fundus imaging.** Optos widefield photo taken 5 days after discharge (A) with corresponding autofluorescence photo (B) showing subtle circumferential hyperfluorescent areas in the temporal periphery (yellow arrows) that demarcate areas that became atrophic with pigmentary changes ∼1 month (C, D) and ∼4 months (E, F) after discharge, which are believed to be Amalric choroidal infarcts. Also note resolution of the superonasal retinal whitening and preretinal hemorrhages (blue arrows) over time. (For interpretation of the references to color in this figure legend, the reader is referred to the Web version of this article.)

At a follow-up visit 5 days later, visual acuity had worsened to 20/250 ph NI, presumably from worsened corneal edema in the setting of the patient's likely anterior segment ischemia. However, at visits ∼1 month and ∼4 months later, the corneal edema and ptosis resolved, and visual acuity improved to 20/100, pinhole 20/60-1. Repeat Optos images showed gradual resolution of the superonasal retinal whitening and preretinal hemorrhages (Fig. 2). However, they also showed the development of large, wedge-shaped areas of pigmentary changes/atrophy in the temporal periphery. These areas are believed to represent Amalric triangular choroidal infarcts, as choroidal ischemia is a known sequela of cosmetic filler-induced ophthalmic artery occlusion.^5^^,^^7^^,^^8^ They are currently being monitored and, given their location, are not expected to have a clinically significant impact. Remarkably, a manifest refraction was performed ∼9 months after the inciting event, which revealed a left eye best-corrected visual acuity of 20/25-2.

## 3 Discussion

We report a unique case of vision recovery after ocular massage in the setting of cosmetic filler-induced ophthalmic artery occlusion. However, it should be noted that there were multiple other, possibly contributing factors. First, immediately after the patient's vision loss was recognized, prior to the patient presenting to the emergency department, hyaluronidase had been administered with an injection to the same area. Nevertheless, this is unlikely to have helped, as the chance of a dermal or subcutaneous injection entering and refluxing up an artery, as had presumably occurred with the filler injection, is extremely low, and it would have been extraordinarily improbable for it to have happened a second time in order for the hyaluronidase to have reached the areas occluded by filler. This is compounded by the fact that blood flow tends to be redirected away from sites of occlusion, and, due to the high risk for thrombosis at sites of occlusion, hyaluronic acid dissolution by hyaluronidase is unlikely to fully clear obstructions. Furthermore, hyaluronidase is unlikely to be able to permeate blood vessels from the outside, as retrobulbar injection of hyaluronidase, which would bring the hyaluronidase in direct proximity of the blood vessels harboring the plaques, has proven ineffective in multiple studies.^9^, ^10^, ^11^

That said, the patient also received hyperbaric oxygen treatments, which could have also played a role in her vision recovery. Notably, although the patient reported a significant improvement in her visual acuity, with resolution of the dark central scotoma, immediately after ocular massage, visual acuity was not fully reassessed until after the patient had returned from her first hyperbaric oxygen treatment session. Nevertheless, given both the patient's subjective report of vision improvement immediately after ocular massage and her denial of further improvement after the hyperbaric oxygen treatment, the ocular massage is still felt to have been the primary factor in restoring the patient's vision. Still, it should be emphasized that this premise is based off the patient's subjective reporting, not objective data. Hence, the potential contribution of hyperbaric oxygen treatment to her recovery cannot be discounted.

Lastly, vision can rarely improve spontaneously, without intervention, after CRAO. An estimated 1–10 % of patients experience spontaneous vision improvement within 48–72 hours of occlusion, although the degree of improvement in these already rare cases appears to be small, with fewer than 10 % improving by 2–3 or more lines on the Snellen acuity chart.^12^ However, such improvement has never been reported to occur in such an acute manner, and the timing directly coinciding with ocular massage makes this unlikely to have been the case for our patient.

It should also be noted that, although we postulate that the patient had a CRAO which transitioned to a BRAO after ocular massage, the presence of a CRAO was never definitively demonstrated. Given the acuity of the situation, the initial dilated fundus exam was not performed until after ocular massage. Still, even if one had been performed, it may not have aided in the diagnosis, as the classic retinal whitening with cherry red spot seen in CRAO typically takes several hours to develop.^13^ That said, the patient's relative afferent pupillary defect and reported central scotoma are both strongly suggestive of a CRAO. The observed superonasal BRAO would not produce a central scotoma, although formal visual field testing to confirm the central scotoma was not conducted. In addition, relative afferent pupillary defects are uncommon in BRAO, whereas they are the norm for CRAO.^14^ Furthermore, the observed choroidal infarcts, anterior segment ischemia, ptosis, and glabellar skin discoloration all strongly suggest upstream occlusion involving the ophthalmic artery, which would also impede flow to the central retinal artery.

While the evidence for ocular massage as an effective treatment for CRAO remains anecdotal, our case has several unique aspects which may have made ocular massage more efficacious than what is typically observed. First, ocular massage was initiated relatively quickly, ∼1.5 hours after the inciting event. While the amount of time retina tissue is able to survive ischemia is unknown, studies in rhesus monkeys suggest it is between 1.5 and 4 hours,^15^ and the American Heart Association guidelines for the effective time window for treatment of an acute ischemic stroke with thrombolytics is within 3–4.5 hours of stroke onset.^16^ In addition, the ocular massage technique that was employed was highly aggressive, exerting significant and rapid pressure fluctuations on the eye, to the point that a “valsava-like retinopathy” was induced, presumably from the shearing of retinal capillaries.^6^ To our knowledge, this is only the second reported case of a “valsava-like retinopathy” induced by ocular massage, and, like the first case, it did not have a permanent effect on visual acuity.^6^ Notably, there are currently no official guidelines for ocular massage technique in the setting of CRAO, only suggestions advocating a relatively gentle approach consisting of repeated cycles of the application of firm pressure, either with fingers or a Goldmann fundus contact lens, to indent the eye ∼2–3 mm, followed by quick release of the eye.^17^ On the other hand, our technique involved indenting/pushing back the eye ∼5mm and employing rapid circular motions with minimal interruption. It is possible that highly aggressive massage is necessary for maximum efficacy. Furthermore, it is plausible that ocular massage is more effective for certain plaque materials. Notably, firmness and viscosity can vary significantly between different hyaluronic acid products depending on the degree of chemical crosslinking and the filler's “G prime” value, which is essentially a measure of its elastic modulus/firmness.^18^ The filler used in our patient's case (RHA® 2 filler) appears to be softer and more flexible than most based on these properties.^19^ One can speculate that softer hyaluronic acid plaques are more prone to being dislodged and displaced distally during ocular massage than harder hyaluronic acid plaques or the more typical cholesterol plaques seen in atherosclerosis. It would be interesting to investigate how the mechanical properties of a plaque (e.g., stiffness, viscoelasticity) correlate with its ability to mobilize within a vessel.

Regardless, our case suggests that aggressive ocular massage might prove effective in cases of hyaluronic acid filler-induced retinal artery occlusions. Nevertheless, it should be emphasized that the technique remains unproven in terms of efficacy and safety. The development of a valsava-like retinopathy, although largely clinically insignificant in our case, demonstrates that the technique is not entirely benign. Other complications, such as retinal detachment and lens dislocation, which have been associated with chronic aggressive eye rubbing,^20^ may also be possible. Furthermore, as discussed above, our case had multiple limitations. Notably, the timing of the patient's vision recovery being immediately after ocular massage (beyond improvement from hand-motion to at least count-fingers vision) was predicated on the patient's subjective report. In addition, the presence of a CRAO was never definitively demonstrated on exam. Hence, the case should not be interpreted as conclusive evidence for the efficacy of aggressive ocular massage. Significantly more case evidence, ideally combined with laboratory data, is needed before recommendations can be made with respect to use of the technique.

## 4 Conclusion

Retinal artery occlusion is a rare but potentially devastating complication of cosmetic filler injection. Here, we report a case of hyaluronic acid filler injection resulting in acute vision loss accompanied by left glabellar skin discoloration, ptosis, anterior segment ischemia, and choroidal infarcts, altogether suggestive of ophthalmic artery occlusion. Interestingly, the patient experienced a remarkable recovery of vision after ocular massage. While there are other reports of vision recovery after cosmetic filler-induced ophthalmic artery occlusion,^21^^,^^22^ our case is unique in that the recovery appears to be primarily from ocular massage, although the timing of the improvement is largely based on the patient's subjective report. Of note, the ocular massage technique that was employed was highly aggressive, exerting significant and rapid pressure fluctuations on the eye, to the point that a “valsava-like retinopathy” was induced, presumably from the shearing of retinal capillaries. Notably, there are no official guidelines for ocular massage technique, and this report suggests that an especially aggressive technique might improve the likelihood of success. However, the technique may not be entirely benign, and more objective case and laboratory data are needed to assess its safety and efficacy.

## CRediT authorship contribution statement

**Alexander A. Svoronos:** Writing – review & editing, Writing – original draft, Validation, Methodology, Investigation, Formal analysis, Data curation, Conceptualization. **Nathan L. Scott:** Writing – review & editing, Validation, Supervision.

## Patient consent

Personal information that could lead to the identification of the patient is not included within this case report. Consent from the patient to publish the report was therefore not obtained.

## Disclosure statement

The authors have no potential conflicts of interest to disclose.

## Authorship

All authors attest that they meet the current ICMJE criteria for Authorship.

## Funding

No funding or grant support.

## Declaration of competing interest

The authors declare that they have no known competing financial interests or personal relationships that could have appeared to influence the work reported in this paper.
